# Dissociation of microdissected mouse brain tissue for artifact free single-cell RNA sequencing

**DOI:** 10.1016/j.xpro.2021.100590

**Published:** 2021-06-10

**Authors:** Lu Liu, Simon Besson-Girard, Hao Ji, Katrin Gehring, Buket Bulut, Tuğberk Kaya, Fumere Usifo, Mikael Simons, Ozgun Gokce

**Affiliations:** 1 Institute for Stroke and Dementia Research, University Hospital, Ludwig-Maximilian-University LMU, 81377 Munich, Germany; 2 Munich Cluster of Systems Neurology (SyNergy), 81377 Munich, Germany; 3 Institute of Neuronal Cell Biology, Technical University Munich, 80802 Munich, Germany; 4 German Center for Neurodegenerative Diseases (DZNE), 81377 Munich, Germany

**Keywords:** Cell Biology, Cell isolation, Single Cell, Flow Cytometry/Mass Cytometry, Sequencing, RNA-seq, Neuroscience

## Abstract

Single-cell RNA sequencing (scRNA-seq) provides the transcriptome of individual cells and addresses previously intractable problems including the central nervous system’s transcriptional responses during health and disease. However, dissociating brain cells is challenging and induces artificial transcriptional responses. Here, we describe an enzymatic dissociation method for mouse brain that prevents dissociation artifacts and lowers technical variations with standardized steps. We tested this protocol on microdissected brain tissue of 3-week- to 24-month-old mice and obtained high-quality scRNA-seq results.

For complete details on the use and execution of this protocol, please refer to Safaiyan et al. (2021).

## Highlights

• An optimized protocol to obtain artifact-free scRNA-seq from mouse brain tissue
• Prevention of dissociation artifacts by blocking transcription
• Standardized steps lower technical variations in droplet- or plate-based scRNA-seq

## Graphical abstract

**Figure:**
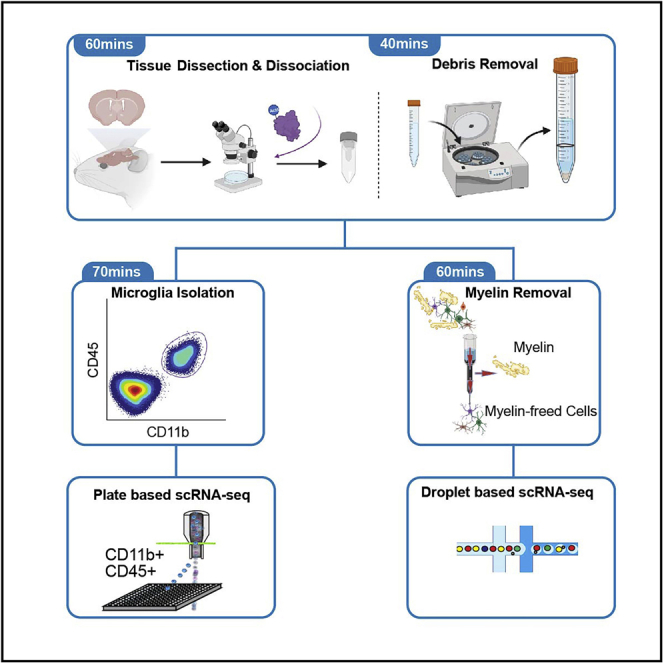

## Before you begin

The protocol below was applied to mouse brains. It hasn’t been tested for other species.

**Figure 1:**
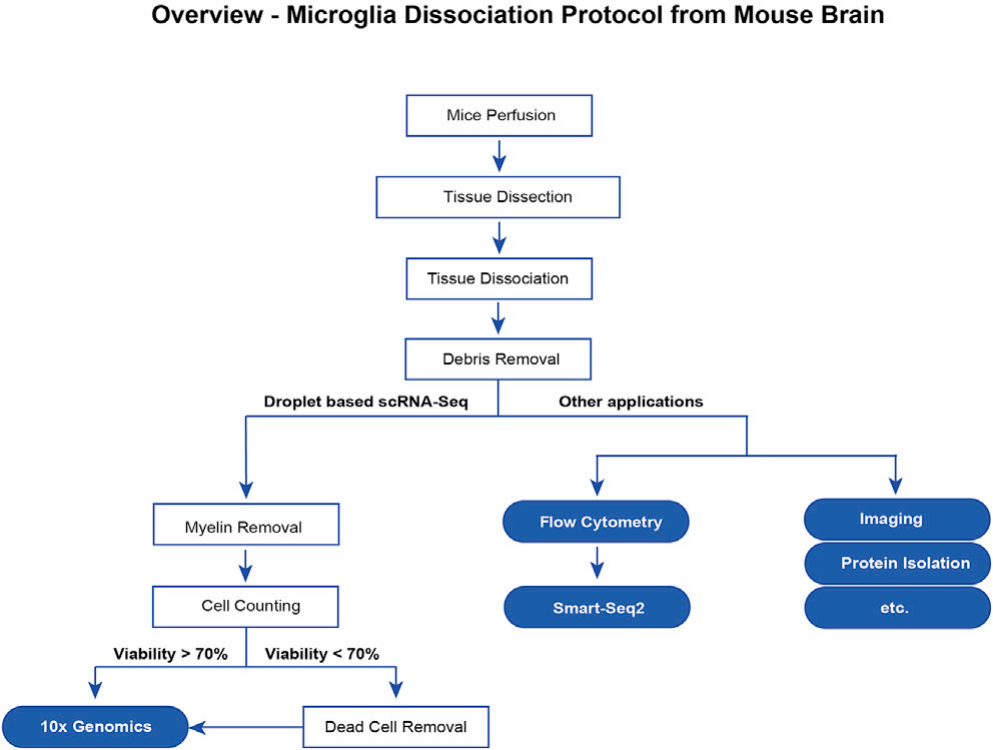
Workflow Graphic summary of the main steps leading to different single cell library preparation methods

The workflow of the whole dissociation process is shown in Figure 1.

### Preparation for tissue dissociation

**Timing: 1 h**

1. If gentleMACS™ OctoDissociator is not available, set water bath temperature to 37°C.
2. Cool down centrifuge (applicable to 50 mL and 15 mL tubes) to 4°C.
3. Prepare the coating buffer by dissolving BSA in DPBS-CMF which might take few hours and filter it through a 20 μm filter.

**Table:** 

3 % (w/v) BSA in DPBS-CMF	0.15 g Bovine Serum Albumin (BSA) + 5 mL DPBS-CMF (for one sample)

***Note:*** Store at −20°C.

4. Thaw Enzyme P and Enzyme A from the “Adult Brain Dissociation Kit” on ice.
5. Using coating buffer, precoat all plastic ware that will be in contact with cells to avoid cells sticking to the plastic (1.5 mL tubes, 15 mL tubes, 50 mL tubes, C Tubes and pipette tips). For coating tubes, fill the tubes with coating buffer and let all the inner walls touch the buffer, afterward remove the buffer from the tube. The removed buffer can be used for coating further plastic ware. Before using tips on cells, draw coating buffer into the pipette tip once and dispense it again, then continue with the cell suspension.
6. Clean dissection tools with 70% ethanol and fill 100/17 mm petri dishes with ice.

## Key resources table

**Table:** 

REAGENT or RESOURCE	SOURCE	IDENTIFIER
**Antibodies**		

Fc-blocking reagent (CD16/CD32 Monoclonal Antibody (93))	Thermo Fisher Scientific	14-0161-82
Rat anti-CD45 (eFluor 450, 30-F11)	Thermo Fisher Scientific	48-0451-82
Rat anti-CD11b (PE/Cy7, M1/70)	Thermo Fisher Scientific	25-0112-82

**Chemicals, peptides, and recombinant proteins**		

DPBS, no calcium, no magnesium\ (DPBS-CMF)	Thermo Fisher Scientific	14190144
Fetal bovine serum	Thermo Fisher Scientific	10270-106
Bovine serum albumin	Roth	8076.4
Actinomycin D	Sigma-Aldrich	A1410
Heparin-Natrium-25000-ratiopharm	Ratiopharm	PZN: 03029843
Trypan Blue Stain (0.4%)	Thermo Fisher Scientific	T10282
7-Aminoactinomycin D (7AAD)	Thermo Fisher Scientific	A1310
Myelin Removal Beads II	Miltenyi Biotec	130-096-731

**Critical commercial assays**		

Adult Brain Dissociation Kit, mouse and rat	Miltenyi Biotec	130-107-677
Dead Cell Removal Kit (optional)	Miltenyi Biotec	130-090-101

**Deposited data**		

Movie: Microdissection White-Grey Matter	This paper	https://dx.doi.org/10.17632/tz3nkpzwhc.1

**Experimental models: organisms/strains**		

Mouse: C57BL/6J	Janvier Labs	N/A

**Software and algorithms**		

FlowJo™ software, version 10	BD	https://www.flowjo.com
Sony SH800 Cell Sorter Software, version 2.1	N/A	N/A
Custom gentleMACS™ Octo Dissociator program	This paper	https://dx.doi.org/10.17632/tz3nkpzwhc.1

**Other**		

50 mL High Clarity PP Centrifuge Tube	Falcon	352070
15 mL High Clarity PP Centrifuge Tube	Falcon	352097
20 μm Filter	Roth	KC72.1
70 μm Filter	Falcon	352350
30 μm Filter	Sysmex	04-0042-2316
FACS cell strainer	Falcon	10585801
Dissection microscope	Zeiss	Stemi 305
Fluorescence microscope	Leica	DMi 8
Forceps	FST	91115-10
Centrifuge	Thermo Fisher Scientific	N/A
Cell counter	Bio-Rad	TC20
GentleMACS™ Octo Dissociator with Heaters	Miltenyi Biotec	130-096-427
C Tubes GentleMACS™	Miltenyi Biotec	130-096-334
LS Columns	Miltenyi Biotec	130-042-401
MACS MultiStand	Miltenyi Biotec	130-042-303
100/17 mm Petri dish	Thermo Fisher Scientific	150350
60/15 mm Petri dish	Greiner Bio-One	628161
Dead Cell Removal Kit	Miltenyi Biotec	130-090-101

## Materials and equipment

Buffer X, Enzyme P, Buffer Y and Enzyme A are from the Adult Brain Dissociation Kit

**Table:** Enzyme Mix 1 (values for one sample)

Reagent	Final concentration	Amount
Buffer X	n/a	1898μL
Enzyme P	n/a	50 μL
Actinomycin D (45 mM)	45 μM	2 μL
**Total**	**n/a**	**1950 μL**

***Note:*** Enzyme Mix 1 can be directly prepared freshly in a C tube and stored on ice until ready to use (Figure 2A).

**CRITICAL:** Actinomycin D is toxic and must be handled with care. Wear gloves and avoid any skin contact.

**Table:** Enzyme Mix 2 (values for one sample)

Enzyme A in Buffer Y	20 μL Buffer Y + 10 μL Enzyme A

**Figure 2:**
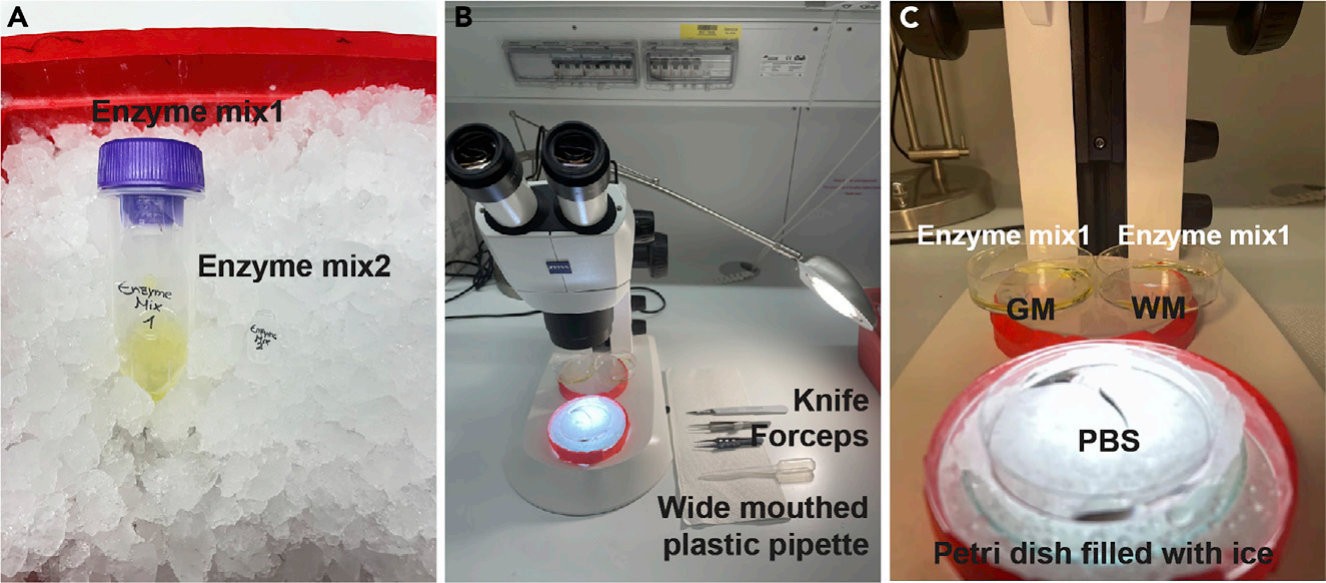
Images of preparation (A) Images of the set up before the experiment: the tubes harboring the prepared enzyme mixture. (B) Images of the set up for dissecting brain tissue. (C) Close up of the set up for dissecting mouse brain tissue.

***Note:*** Prepare freshly in a separate tube and store on ice until ready to use (Figure 2A).

**Table:** Perfusion Solution (values for one sample)

DPBS-CMF with Heparin	30 mL DBPS-CMF + 2 UI/mL Heparin

***Note:*** A diluted solution of heparin inhibits blood clot formation and preserves the patency of the vascular system. Prepare freshly and store on ice until ready to use.

**Table:** Loading buffer (only required for Drop-seq based scRNA-seq (10× Genomics), values for one sample)

0.04% (w/v) BSA in DPBS-CMF	15 mL DPBS-CMF + 0.2 mL coating buffer

***Note:*** Prepare freshly and store on ice until ready to use.

**Table:** Flow cytometry buffer (only required for flow cytometry, values for one sample)

2% (w/v) FBS in DPBS-CMF	12.5 mL DPBS-CMF + 250 μL FBS

***Note:*** Prepare freshly and store on ice until ready to use.

**Table:** Blocking buffer (only required for flow cytometry, values for one sample)

Fc Receptor blocking reagent in flow cytometry buffer	50 μL flow cytometry buffer + 0.5 μL Fc Receptor blocking reagent (1:100)

***Note:*** Prepare freshly and store on ice until ready to use.

**Table:** Antibody staining mixture (only required for flow cytometry, values for one sample)

Antibodies in flow cytometry buffer	50 μL flow cytometry buffer + 0.5 μL eFluor 450 anti-CD45 (1:100) + 0.5 μL PE/Cy7 anti-CD11b (1:100)

***Note:*** Prepare freshly and store on ice until ready to use.

## Step-by-step method details

### Mice perfusion and dissection

**Timing: 30 min / mouse**

We routinely performed this protocol for young and old (3 weeks to 24 months old) mouse tissue from whole brain, or from dissected regions such as prefrontal cortex, hippocampus and white matter. While we have not tested this protocol on every age and region of the mouse brain, we believe that it is capable of isolating single cells from all regions and ages of mouse brain samples. We recommend performing a pilot experiment to determine the number of cells that can be obtained from the region of interest with one animal, then scale up accordingly. For the white matter tissue and prefrontal cortex (gray matter), we generally pool white matter dissected tissue from 2–3 mice together in one C tube. Troubleshooting 4

1. Tissue harvesting
  a. Anesthetize the mice and transcardially perfuse with 20 mL cold perfusion solution for 3–5 min as described by Nicolás-Ávila et al., 2021. Troubleshooting 1
  b. Perform cervical dislocation, cut the head and carefully free the brain from the skull. A proper transcardial perfusion results in white not pink colored brain. Transfer the brains in 15 mL tubes filled with ice-cold DPBS-CMF.
2. Dissection of targeted brain region. (Methods video S1: Microdissection of White and Grey matter of mouse brain, related to step 2.)
  a. Set up the dissection workplace to keep tissue ice cold during dissection as shown in Figures 2B and 2C. Perform the dissection in a petri dish (60/15 mm or similar), filled with cold DBPS-CMF, and on ice. Microdissected tissue are transferred to a new petri dish (60/15 mm or similar), filled with cold 1 mL of Enzyme Mix 1, and on ice. Prepare separate petri dish for each target regions (e.g one carrying white matter tissue and the other gray matter tissue Figures 2B and 2C). An explanatory Movie describing the mouse brain dissection of white and gray matter of the prefrontal cortex is provided (Methods video S1). The total dissection of all samples should be as quick as possible, and the total duration should be less than 2 hours. To prevent transcriptional responses, brains should be kept cold on ice and dissected tissue immediately placed in ice cold Enzyme Mix 1 with transcription inhibitor actinomycin D (Wu et al., 2017).

**CRITICAL:** All steps should be done on ice except the enzymatic digestion.

Methods video S1. Microdissection of White and Grey matter of mouse brain, related to step 2

### Tissue dissociation

**Timing: 30 min**

In this step the tissue is dissociated using the automized gentleMACS™ Octo Dissociator with a customized program, which decreases technical variation compared to manual mechanical dissociation.

3. Add 30 μL of Enzyme Mix 2 to each C Tube.
4. Tightly close the C Tubes and attach them upside down onto the gentleMACS™ OctoDissociator. Tap the tubes to move tissue on the walls into enzyme mix.
5. Run the “15 min dissociation program” (Figure 3A)

**Figure 3:**
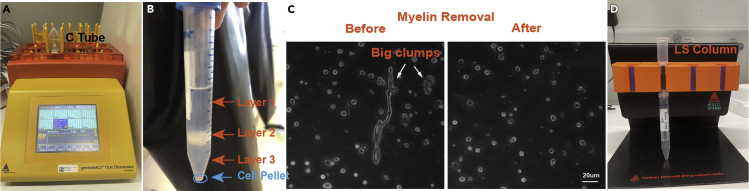
Images of critical steps (A) Dissociation: Images of the C tube attached to the gentleMACS™ Octo Dissociator with Heaters. (B) Debris removal: Images of the two layers (layer1+layer2) to be removed after first centrifugation in the debris removal process. (C) Images of the cell suspension before and after myelin removal process. (D) Myelin removal: Images of the set up for the myelin removal process.
6. Upon completion of the program, detach the C Tubes from the gentleMACS™ Octo Dissociator.
7. Place 70 μm filters on 50 mL tubes and pre-wet the filters with 500 μL of cold DPBS-CMF. Filter the dissociated tissue samples by pouring the dissected tissues onto the filters.
8. Add 9.5 mL of cold DPBS-CMF into each C Tubes, shake them gently and add the liquid to the respective filter to collect remaining tissue fragments and cells.
9. Discard the 70 μm filter and centrifuge cell suspension at 300 × *g* for 10 min at 4°C. Aspirate supernatant slowly and avoid disturbing the cell pellet.

**CRITICAL:** The gentleMACS™ OctoDissociator is using a custom shortened program for scRNA-seq, which may decrease cell yield, but is half of the duration which, as a result, limits transcriptional responses to dissociation.

Original gentleMACS™ Program for dissecting brain tissue:

20–100 mg: 37C_ABDK_02

>100 mg: 37C_ABDK_01.

We use a custom gentleMACS™ Octo Dissociator program (Supplementary file 1) optimized for microdissected tissue for scRNA-seq.

Instruction on how to install the program in the gentleMACS™ Octo Dissociator:

• First create a folder named “GM8” in a USB stick, and copy the program file into this folder.
• Then connect the USB stick to the device
• Click in the USB stick folder logo that appears on the left side of the screen
• Select the program file “37C_cus_NT_15_1” by clicking on the checkbox on the right side
• Click the Save button. A window will appear asking where to save the file. In this window click on the folder logo to find the folder where to save the file. Click ok.
• Open the destination folder to ensure that the transfer was successful. For more details, please refer to gentleMACS™ Octo Dissociator with Heaters Manuals section 4.5.1.

***Note:*** The program “37C_cus_NT_15_1” consists of three incubations at 37°C of 4.5 min each and in between each incubation there is mechanical dissociation. First mechanical dissociation is set at 100 rpm and the last two are set at 300 rpm. The temperature is set to 37°C during the whole program. The total duration of the program is 15 min.

***Note:*** If the gentleMACS™ Octo Dissociator is not available in the lab, preheat the tissue in the Enzyme Mix 1 at 37°C for 15 min in a water bath at 37°C and proceed with manual dissociation on ice. For the manual dissociation, tissues should be triturated by three separate flame-polished Pasteur pipets with successively smaller openings (Preparation of 3 Pasteur Pipets and their application are described by (Bordt et al., 2020) and continue with Step 10.

### Debris removal

**Timing: 40 min**

In this step, cell debris are removed by a gradient centrifugation to obtain a single-cell suspension.

10. Resuspend the cell pellet gently in 1550 μL of cold DPBS-CMF and transfer the cell suspension to a 15 mL falcon. **Do not vortex cell suspensions!**
11. Add 450 μL of cold Debris Removal Solution from the “Adult Brain Dissociation Kit”.
12. Mix well by using a 1000 μL pipette.
13. Overlay very gently and slowly with 2 times 1 mL cold DPBS-CMF by using a 1000 μL pipette.
14. Centrifuge at 4°C and 3000 × *g* for 10 min with acceleration 9 and brake 9.
15. Three layers will form (Figure 3B). Aspirate the two top layers completely with a vacuum pump.
16. Fill the tube up to 5 mL with cold DPBS-CMF.
17. Gently invert the tube three times. **Do not vortex!**
18. Centrifuge at 4°C and 1000 × *g* for 10 min with acceleration 9 and brake 9. Aspirate supernatant completely and avoid disrupting the cell pellet.

**CRITICAL:** After the debris removal part, the next step depends on which method is chosen by the researchers.

Droplet based scRNA-seq: please follow the tiles with “for 10× Genomics”, continue with step 19.

Plate based scRNA-seq: please follow the tiles with “for flow cytometry”, continue with step 32.

### Myelin removal (for 10× Genomics)

**Timing: 60 min**

The myelin removal part is necessary to remove myelin and big clumps, which can block the 10× loading chip. This step also removes most of the oligodendrocytes. A comparison of cell suspensions under light microscopy, before and after the myelin removal part, is shown in Figure 3C.

19. Resuspend the cell pellets in 270 μL of loading buffer and add 30 μL Myelin Removal Beads II. Mix well with the pipette, but do not vortex. Incubate for 15 min at 4°C.

***Note:*** These specific volumes are sufficient to remove myelin as well as most of the oligodendrocytes from cell suspension of grey matter and white matter from three adult animals. Adjust the bead volume for the target brain region according to the data sheet.

20. Wash the cells by adding 10 times the labeling volume (in this case: 2700 μL of loading buffer) and centrifuge at 300 × *g* for 10 min.
21. During the centrifugation time, place the appropriate number of LS Column (according to manufacturer of Myelin Removal Beads II) in the magnetic field of a suitable MACS Separator (Figure 3D).

***Note:*** From now on, preferably work in a 4°C cold room until step 26 is done.

22. Rinse the column with 3 mL loading buffer.
23. After the centrifugation, aspirate the supernatant completely.
24. Add 1000 μL of loading buffer for each LS Column and resuspend the cells.
25. Apply the cell suspension onto the LS column. Collect the unlabeled cells that pass through. This is the myelin freed sample. **Do not trash!**
26. Wash the column with 2 times 1 mL of loading buffer. Collect the total effluent, since it still contains the myelin freed sample. Only add new buffer for washing when the column reservoir is completely empty.
27. Filter the collected cell suspension through a 30 μm filter (prewetted with 500 μL DPBS-CMF)
28. Centrifuge for 10 min at 300 × *g* at 4°C.

### Cell counting (for 10× Genomics)

**Timing: 10 min**

The cell number and viability are counted to confirm that the requirements for loading the sample on a 10× genomics chromium controller are fulfilled. After a final filtering step, the single-cell suspension is ready to be loaded on the 10× Genomics Chip.

29. After centrifugation, discard the supernatant and gently resuspend gray matter cell pellets in 120–160 μL of loading buffer (for 10× Genomics), and white matter cell pellets in 60–80 μL of loading buffer.

***Note:*** When doing the experiment for the first time, start the resuspension with a quite low volume, so the cell density can be adjusted to 10**×** Genomics requirements accordingly.

30. Take 10 μL of the cell suspension and mix thoroughly with 10 μL of trypan blue. Load the mixture into a manual cell counting slide or an automated cell counter such as Bio-Rad T20 to determine the live and dead cells concentrations. Adjust the cell concentrations according to the recommendation of the 10× Genomics protocol. Troubleshooting 2 and 3

**CRITICAL:** If the protocol is done fast enough, the live cell ratio should be above the 70% recommended in the 10**×** protocol. If cell survival is low, optimize the duration of the protocol and make fresh buffers.

**Figure 4:**
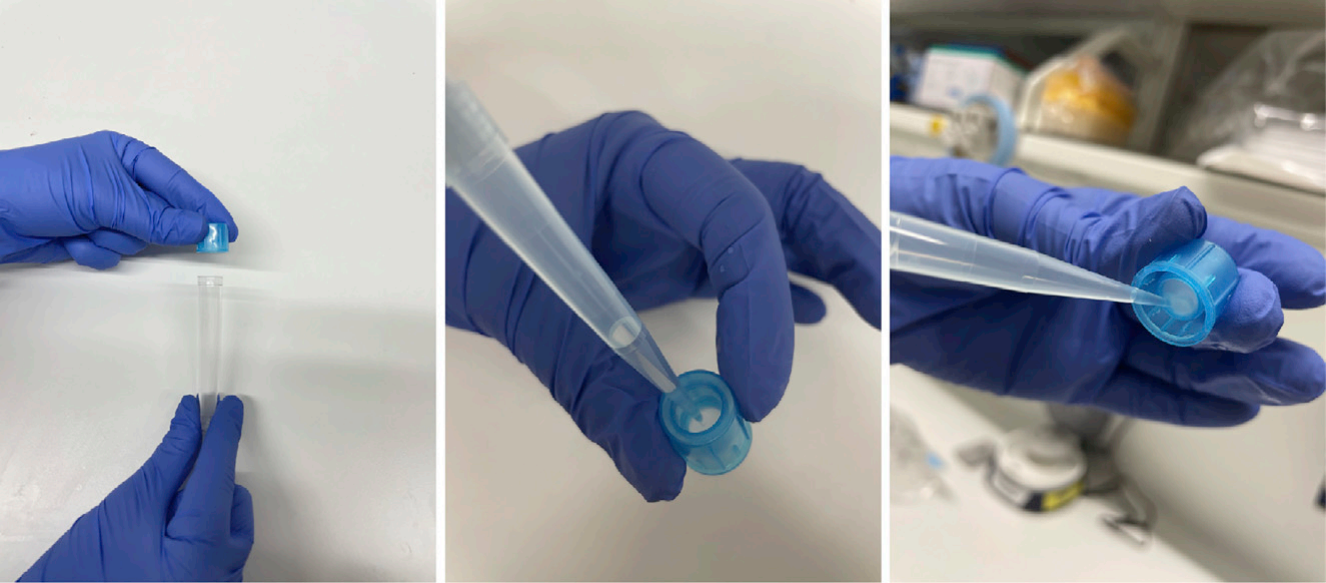
Images on using the flow cytometry cell strainer to filter low volume cell suspension

31. Before loading, filter the low volume cell suspension through a 45 μm filter (flow cytometry cell strainer).
  a. For filtering, detach the cap of a flow cytometry cell strainer from the tube and discard the tube. Pre-wet the 45 μm filter in the cap with 1000 μL of DPBS-CMF.
  b. Then carefully add the sample on top of the filter. On the bottom side of the filter, a drop will form.
  c. Carefully draw the hanging drop from the bottom of the filter with a 1000 μL pipette (Figure 4)

**CRITICAL:** This filtering approach will free the small volume sample from debris gently with minimal loss.

***Note:*** The data of single cell RNA-seq can be analyzed with Seurat (Stuart et al., 2019), or other available platforms.

### Isolation of microglia from cell suspensions (for flow cytometry)

**Timing: 70 min**

If the plate based scRNA-seq is required, after finishing step 18, please directly continue with step 32.

The single cell suspension is labeled with antibodies for flow cytometry, to finally gate microglia.

32. After the debris removal part, resuspend the cell pellets in 1 mL DPBS-CMF and filter the collected cell suspensions through a 30 μm filter (prewetted with the 500 μL DPBS-CMF) into 15 mL falcon. Wash the filter with 2 times 1 mL of DPBS-CMF and collect the total effluent.
33. Centrifuge for 10 min at 300 × *g* at 4°C and discard the supernatant.
34. For each flow cytometry sample, resuspend the cell pellet (up to 10^7^ cells) gently in 50 μL of blocking buffer. Incubate the samples for 10 min at 4°C.
35. Meanwhile, prepare the desired antibody staining mixture.

**CRITICAL:** All antibodies should be tested beforehand with unstained and single stained controls. We recommend applying the compensation process on the flow cytometry machine using control samples (unstained control samples and single stained control samples).

After the 10 min incubation, add 50 μL of the prepared antibody staining mixture to each sample. Incubate the samples for 20 min at 4°C in the dark.

**Figure 5:**
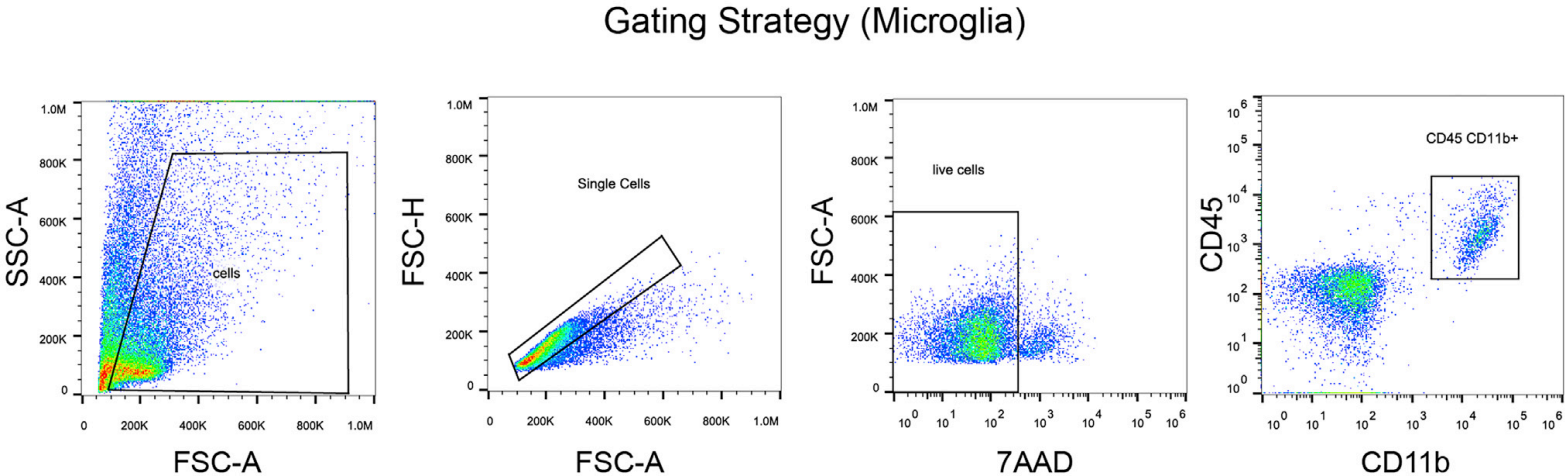
Full gating strategy for the selection of single live (7AAD-) microglia (CD45+CD11b+) of white matter from a 24-month-old mouse brain

36. Then add 3 mL of DPBS-CMF to each sample.
37. Centrifuge for 10 min at 300 × *g* at 4°C.
38. Carefully remove the supernatant by a vacuum pump and/or by pipettes. Remove as much liquid as possible.
39. Add 500 μL of flow cytometry buffer to resuspend the pellet.
40. Put 7-AAD (final concentration: 25 μg/mL) in each sample and incubate for 5 min at 4°C in the dark (7-AAD can be used for the exclusion of non-viable cells in flow cytometry analysis).
41. Continue cell sorting in a single cell sorting mode with a flow cytometer (e.g., Sony SH800) selecting the proper gates for each antibody.
42. Standard forward scatter height versus area criteria were used to discard doublets and capture singlets. A membrane impermeant dye (7-AAD) is used for negative selection to remove dead cells. For the gating strategy for microglia, see Figure 5. Troubleshooting 2 and 3

***Optional:*** Sort the CD45+CD11b+ single cells into 96 or 384 well plates filled with lysis buffer for a plate-based scRNA-seq method such as Smart-Seq2.

## Expected outcomes

Using our optimized protocol, for 10× Genomics, we can achieve approximately 60,000 - 120,000 live cells from white matter of three mice. However, the result can be influenced by the size of the dissected tissue and the age of the mice. Using our optimized protocol, for flow cytometry we can achieve approximately 600–800 sorted single-cell microglia from white matter per mouse. The sorter can exclude most of the myelin and dead cells. These cell suspensions can be alternatively applied to imaging and protein isolation.

## Limitations

For 10× Genomics, as white matter is composed of large amounts of myelin, a myelin removal step is necessary to avoid large myelin clumps that potentially block the capillaries of the 10× chromium loading chip. However, most of the oligodendrocytes are also removed as they attach to the myelin sheath. If oligodendrocytes are needed for further analyses, the myelin removal step can be replaced with sorting the live cell population.

In flow cytometry, the shear stress of sorting can activate the cells and lower cell survival rate if functional assays are planned.

## Troubleshooting

### Problem 1

High number of blood cells in analysis data. This could be due to insufficient transcardial perfusion or bleeding in the tissue.

### Potential solution

Check color of the dissected tissue. If the tissue has abnormal blood clots or appears pinkish, improve transcardial perfusion. The red blood cell lysis solution from the Adult Brain Dissociation Kit can be used to lyse red blood cells. However, if brain resident immune cells are of interest, we recommend improving perfusion to eliminate most of the immune cells in the circulating blood. (step 1 in step-by-step method details).

### Problem 2

Low cell viability.

### Potential solution

All steps of the protocol are optimized to increase viability. Maintaining all solutions fresh and conducting this protocol smoothly can improve viability. If problem persist, remove dead cells using the Dead Cell Removal Kit (step 30 in step-by-step method details).

### Problem 3

Low number of cells.

### Potential solution

If dissected tissue is small, the cell pellet might be barely visible after centrifugation and might be lost due to improper resuspension or accidently aspirated. Cell loss could be reduced by careful aspiration and resuspending carefully with a pipette multiple times (steps 9, 15, 18, 23, 29, 33, 38 in step-by-step method details). If those steps are not sufficient, try to increase the amount of starting material by increasing animal numbers. Also watch if the cell strainer is blocked, and prevent cells to pass through.

### Problem 4

There is a limited number of animals which can be used for the experiment and they cannot be pooled together to achieve enough target tissue.

### Potential solution

This can be solved by performing the dissociation on each sample separately first and then apply cell hashing (Stoeckius et al., 2018) or MULTI-seq (Gehring et al., 2020) methods to label each sample before pooling them together (step 2 in step-by-step method details).

### Problem 5

Analysis of flow cytometry isolated microglia scRNA-seq results identifies non-microglial cell types (for Flow Cytometry approach).

### Potential solution

If the non-microglial cell types specifically originated from one experimental condition, this might be due to up regulation of microglial markers in other cell types. Otherwise, this indicates a problem with the flow cytometry. Before the experiment, performance of each antibody and dye should be validated. In the experiment, include all controls for flow cytometry:

Negative (unstained) controls: determine background staining.

Single stained controls: validate the performance of each antibody or dye.

Fluorescence minus one (microglia) controls: discriminate target cell type by omitting the antibodies of target cells type.

If problem persist, consider enriching microglia by magnetic bead-based selection to reduce the contamination (steps 35 and 42 in step-by-step method details).

## Resource availability

### Lead contact

Further information and requests for resources and reagents should be directed to and will be fulfilled by the lead contact, Dr. Ozgun Gokce.

### Materials availability

This study did not generate new unique reagents or mouse lines.

### Data and code availability

The accession number for the single-cell RNA-seq data reported in this paper is GEO: GSE166548. The custom gentleMACS™ OctoDissociator program can be downloaded at https://dx.doi.org/10.17632/tz3nkpzwhc.1 The graphical abstract is created with BioRender.com.
